# Cost-utility analysis of palliative care in patients with advanced cancer: a retrospective study

**DOI:** 10.1186/s12904-021-00816-0

**Published:** 2021-08-11

**Authors:** Hao Wu, Ping Lin, Shujuan Yang, Wei Zhang, Wenjuan Tao

**Affiliations:** 1 Institute of Hospital Management, West China Hospital, Sichuan University, Chengdu, 610041 Sichuan China; 2 West China School of Public Health and West China Fourth Hospital, Sichuan University, Chengdu, 610041 Sichuan China

**Keywords:** Palliative Care, Conventional Anticancer Treatments, QoL, QALYs, Cost-Utility Analysis

## Abstract

**Background:**

Aging population and other factors have led to a rapid rise in cancer incidence in China. However, under the influence of traditional perception of diseases, deaths and economic factors, many patients who are unresponsive to radical treatment are still adherent to excessive and unnecessary treatment, which may lead to poor quality of life (QoL) and increase unnecessary medical burden.

**Aim:**

Compare the difference of the quality of life and cost-utility value between patients who received palliative care (PC) and patients who were adherent to conventional anticancer treatment (CAT) and provides empirical evidence of clinical and economic value for hospital-based PC.

**Methods:**

Chinese Quality of Life Questionnaire (CQLQ) Scale was used to collect advanced cancer patients’ QoL on admission and discharge days. Paired and independent samples’ statistical analysis were used to compare inter- and intra- QoL between PC and CAT group. Delphi and Analytic Hierarchy Process were used to weight QoL scores and converted the QoL to quality-adjusted life years (QALYs). Propensity Score Matching (PSM) for 1:1 was used to compare average hospitalization expenses between two groups. The expense per QALYs was used for Cost-Utility analysis between the two treatments.

**Results:**

A total of 248 hospitalized patients diagnosed with metastatic disease at stage IV were recruited from West China Fourth Hospital between January 2018 and August 2018, including 128 patients receiving PC and 120 patients receiving CAT. Although both treatments had positive effects on improving QoL for patients, the QoL in the PC group were significantly higher than that in the CAT group (55.90 ± 18.80 vs 24.00 ± 8.60, t = 7.51, *p* < 0.05). The QALY (days) of pre- and post- treatment increased by 55.9 and 24.0 days in PC and CAT group respectively. Compared average hospitalization expense in 613 pairs of advanced cancer inpatients after PSM 1:1, the per capita expense of PC group was higher (13,743.5 ± 11,574.1 vs 11,689.0 ± 8876.8, t = 3.44, *p* < 0.05), while each unit of QALYs paid by PC group was only 50% of that paid by those receiving CAT.

**Conclusions:**

PC played a positive role in improving the QoL for patients diagnosed with advanced cancer and alleviating economic burdens of both patient families and the society from the viewpoint of cost-utility. Our findings imply that PC should be recognized as a proactive care model in China that helps patients with some terminal diseases.

## Background

The aging population, environmental pollution, unhealthy lifestyles and behavioral habits, and more inducements have led to the continuous rise in the incidence and mortality of cancer in China, which has the top rank in the world [1]. Additionally, because many Chinese people lack cancer prevention consciousness and early cancer-screening covers limited cancer categories, the proportion of patients diagnosed with intermediate and advanced cancer at the time of their initial diagnosis is much higher than the world average ratio [2]. Currently, the rapid development of molecular targeted therapy for cancer, immunotherapy, gene therapy and other technologies contribute to improve the effectiveness and efficiency of treatment procedure. However, for advanced cancer patients who are unresponsive to radical treatment, adherence to anti-cancer therapy not only fails to effectively prolong patients’ survival time, but also aggravates their pains brought by excessive and unnecessary treatments, even brings a heavy economic burden to both families and the society under the co-payment mechanism [3–6]. This vulnerable population is worthy of attention and should be properly guided to seek adequate medical treatment and make reasonable medical decisions.

Palliative care (PC) emerged with the establishment of the modern medical model as a burgeoning branch of clinical medicine which had received wide attention in many countries. Different from conventional anticancer treatment (CAT) which focuses on killing and inhibiting cancer cell reproduction and metastasis through chemotherapy, radiotherapy, surgery, and/or hormone therapy; rather, the goal of PC is to anticipate, prevent, and reduce suffering through patient- and family-centered health care. To help patients and their families better understand prognosis and treatment options, clarify goals of care, and assist in planning for disease progression, the hospital-based interventions are usually designed according to patient's needs, values, beliefs, and cultures by an interdisciplinary PC team [7–10]. However, for a long time, China’s medical practice of “Focusing on Treatment while Ignoring Prevention” has led to the coverage rate of early cancer-screening is low [11] and the medical resources invested in CAT are far greater than those invested in PC program at government level [12]. In addition, medical institutions paid little attention to PC at the institutional level, and there was a lack of adequate knowledge of PC among patients and their families at the public level, which led to oncologists and healthcare professionals were not actively in introducing PC to patients with necessary indications, and patients and their families had a bias against to PC [13]. Thus, the processes of PC system construction and development were slow, and the contradiction between supply and demand was obvious [14].

The elderly tends to be at high risk of advanced cancer and terminal diseases, with the aging of China’s population, the increasing demands of a large group of cancer patients have intensified the urgency of empirical evidence of hospital-based PC's clinical and economical value [15, 16]. Previous studies have shown PC has advantages in improving QoL and reducing economic burden compared to CAT. For example, Zimmermann used clusters randomized trials and found significant improvements in the QoL of patients receiving early PC interventions compared with those who received usual care [17]. A meta-analysis concluded that early PC intervention may decrease symptom intensity and improved the QoL of patients [18]. H. Zhuang et al. indicated that early PC could be a clinically meaningful and feasible care model for improving patient’s QoL [19]. But few previous studies have ascertained the PC utility both from quality and cost perspectives in China. China has a special environment in the field of PC, such as traditional cultural background (regard "Palliative Care" as "Abstention therapy”), traditional Chinese medicine (such as herb and acupuncture) and special medical pricing system (medical price follows government guidelines). These characteristics require us to further explore PC as a proactive care model, especially on the background of the Quality of Death Index for Chinese residents is low in the global ranking [20] and medical burden constantly soaring [21]. Therefore, this research aimed at making a comparative study of QoL, quality-adjusted life years (QALYs), and medical expenses of advanced cancer patients receiving PC and CAT, and evaluate the effectiveness of PC in helping patients make appropriate medical decisions on the perspective from improving QoL and reducing medical burden.

## Materials and methods

### Study patients

The study protocol was approved by the Ethics Committee of West China Fourth Hospital of Sichuan University. The PC unit of the hospital, founded in 1996, is the “International Palliative Care Cooperation Center”, which belongs to the Oxford International Palliative Medicine Convening Center of the WHO. In addition to providing professional PC services, the hospital also has set oncology beds for meeting the needs of usual anticancer patients.

A questionnaire survey on advanced cancer patients’ QoL was conducted in 2018. We recruited 300 participants from hospitalized patients who were diagnosed with advanced cancer (including breast, prostate, lung, colorectal and other malignancies) and hospitalized in Palliative Care and Oncology Department on their admission day and discharge day from January 1, 2018, to August 31, 2018. Eligible participants had to meet the following criteria: patients who were diagnosed with metastatic disease at stage IV with moderate or higher pain symptoms (Pain score ≥ 5), an estimated survival time of more than 1 month and less than or equal to 12 months, an age of 18 years and above, and normal expression. While, patients with an estimated survival period of fewer than 1 month, with mental confusion and cognitive and mental disorders, and who did not complete the questionnaires were excluded.

### Grouping and Intervention

Palliative Care Department and Oncology Department are two relatively independent medical units in the hospital, there are significant differences between in terms of patient access criteria, the path of therapy, expectations and post healing goals. PC is mainly applied to the treatment of some life-limiting diseases, and focusing on effective management of pain and other distressing symptoms to reduce patients’ and their families’ suffering and to support the best possible QoL in the path of therapy. While appropriate CAT performed in oncology Department is aligned with stated patient goals and priorities, through the selection of chemotherapy, radiation therapy, immunotherapy and other appropriate treatments, to help patients control their disease and prolong their lifetime.

**Fig. 1:**
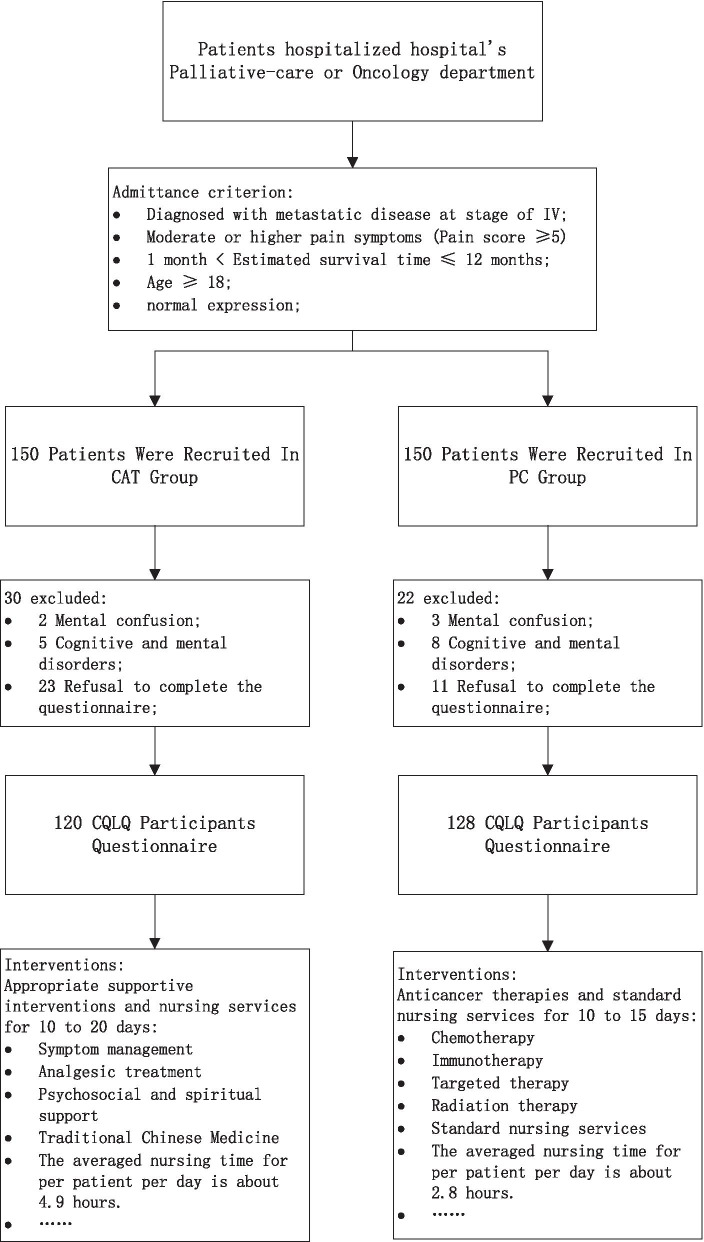
Questionnaire inclusion and exclusion criteria

The choice of therapy method largely depends on the doctor’s opinion and patients' and their families' willingness. Typically, oncologists will follow the medical guidelines and recommend eligible patients who have a life expectancy of 6–12 months referrals to PC unit. An interdisciplinary PC team will work together with the oncologist to provide consultative and assessment according to the patient's situation, and deciding whether to transfer to the palliative ward for supportive care. But some patients and their families may refuse or delay transfer to PC and encourage efforts to cure over the alleviation of suffering for cultural values, economic or other reasons. Thus, we distinguish participants in the PC group or in CAT group according to whether the patient was normally referred from Oncology Department to the regimen provided by PC team, as shown in \* MERGEFORMAT Fig. 1.

Patients in the PC group usually received 10 to 20 days of supportive interventions during hospitalization. PC doctors make appropriate supportive intervention plans according to the patient's specific situation, including analgesic treatment, symptom management, palliative sedation, spiritual support, acupuncture and so on. Specialist nurses provide patients with lymphedema massage, psychosocial and spiritual support, comfort care, health education and other supportive care services, the average nursing time for per patient per day is about 4.9 h. While patients in the CAT group usually received 10 to 15 days of anticancer therapies including chemotherapy, immunotherapy, targeted therapy and standard nursing services during their hospitalization. Although CAT patients would also receive symptom management, palliative sedation and other PC services according to the needs of their situation, they could not receive a systematic PC intervention compared with patients in the PC group. The average nursing time is about 2.8 h for per patient per day.

### Questionnaire design and Statistical Analyses

We used the Chinese Quality of Life Questionnaire (CQLQ) scale to collect QoL information of patients. The CQLQ passed reliability and validity test and is a standard questionnaire for patients with all types of cancer in China [22, 23]. The questionnaire has 12 items and three dimensions referring to the patient's physical symptoms, psycho-mental state and social relations. Each item has 5 options corresponding to a score of 1–5 (1 for the worst function and state, and 5 for the best). The questionnaire has a total score of 60 points, with which the QoL was classified to: Extremely Poor ≤ 20; Poor: 21–30; Medium:31–40; Good:41–50; and Excellent:51–60.

Groups were compared with χ^2^ tests for binary data and the quantitative data form a normal distribution. To analyze the effects of two different treatment methods on improving the QoL of patients, we compared the QoL scores of two groups of patients on pre- and post- treatments, and used the paired sample T test to evaluate the difference of therapeutic effect between PC and CAT (α ≤ 0.05). In addition, to compare the differences between the two groups of patients before and after treatment respectively, we used independent sample t test to evaluate the situation of the two groups of patients pre- and post- treatments (α ≤ 0.05). Data were analyzed using R statistical software (V 3.6.1).

### QALYs calculation

A total of 33 experts with more than 10 years of experience in the fields of oncology and PC were invited to conduct a quantitative evaluation of the importance of the indicators in the CQLQ scale. According to the experts' evaluation, we used the analytic hierarchy process software Yaahp (V 12.1) to determine the weight of indexes at two levels and then used the principle of probability multiplication to calculate the QoL Composite Index:

1 $$Utility\;Values\;of\;QoL\:=\:\sum\;index\;scores\;in\;the\;QLQ\;scale\;/\;5\:\times\:index\;weight$$

The CQLQ has been tested for reliability and validity, and patients' survival time was adjusted by the utility value, i.e., the quality-adjusted life years (QALYs). Survival prediction of patients plays an important role in the evaluation of the clinical value. Cui proposed a new prognostic scale, which may help guide physicians in predicting the likely survival time of advanced cancer patients more accurately [24]. A prospective study reported that patients in the early PC group had significantly higher Kaplan–Meier 1-year survival rates than the delayed PC group [25]. Robert et al. introduced PC for patients who had no response to radical therapies and had a life span of less than 12 months [26]. Following the previous literature, from the perspective of encouraging advanced cancer patients to receive early PC intervention and on the assumption that PC does not significantly shorten patients' survival time [27, 28], we used an expected survival time for patients with advanced cancer of 12 months and converted the mean QoL score to QALYs before and after PC and CAT. Independent samples tests were used to examine differences in the increment of QALYs between two groups:

2 $$QALYs\:=\:\sum\;index\;scores\;in\;the\;CQLQ\;scale\:\times\:survival\;time\;of\;patient(12\;months)$$

### Cost-Utility Analysis

To compare the cost and utility difference between PC and CAT, we extracted the hospitalization expenses of patients with advanced cancer admitted by the two departments Palliative Care and Oncology from January 1, 2016, to August 31, 2018, in West China Fourth Hospital. We screened patients with stage IV (T4) or with metastasized (M1) noted in electronic records and divided patients into a PC group and a CAT group and collected the total medical expenses of the patients during their hospitalization, including pharmaceutical, surgery, nursing and imageological and laboratory examination expense etc. To eliminate the impacts of confounding factors such as patient age, sex, length of stay (LOS), and type of illness on medical expenses, we used propensity score matching (PSM) for 1:1 pairing [32, 33]. Then, calculated average medical expenses per hospitalization for both groups.

3 $$Medical\;expenses\;paid\;for\;the\;unit\;utility\:=\:Average\;medical\;expenses\;per\;hospitalization/\;QALYs\;increment$$

QALYs were used as the utility index to compare the medical cost-utility value of patients in the PC and CAT groups.

## Results

### Comparison of QoL

We interviewed patients and their families together with clinical staff. A total of 300 questionnaires were distributed, and 248 valid responses were received, of which 128 were in the PC group and 120 were in the CAT group, yielding a response rate of 86.3%, as listed in Table 1. The average length of stay (LOS) for patients in the PC group was 14.55 (SD = 15.01) days, compared with 12.80(SD = 11.53) days for patients in the CAT group.

**Table 1:** Characteristics of Patient Participants by Treatment Group

Variable		PCs(N = 128)		CATs(N = 120)		P
		**n/**$\overline{{\varvec{x}} }$	**%**	**n/**$\overline{{\varvec{x}} }$	**%**	
**Sex**						0.987
	Male	62	48.44	58	48.33	
	Female	66	51.56	62	51.67	
LOS		14.55	-	12.80	-	-
**Age**						0.008
	≤ 40	4	3.13	9	7.50	
	40—50	18	14.06	21	17.50	
	51—60	33	25.78	50	41.67	
	61—70	36	28.13	17	14.17	
	71—80	26	20.31	17	14.17	
	> 80	11	8.59	6	5.00	
**Education**						0.828
	Less than primary school graduate	5	3.91	2	1.67	
	Primary school	39	30.47	28	23.33	
	Junior school	34	26.56	57	47.50	
	High school	30	23.44	20	16.67	
	College or above	20	15.63	13	10.83	
**Marriage**						0.098
	Unmarried	18	4.06	8	6.67	
	Married	110	85.94	112	93.33	
**Residence**						0.237
	Urban	64	50.00	51	42.50	
	Rural	64	50.00	69	57.50	
**Insurance**						0.339
	UEMI	49	38.28	56	46.67	
	URMI	18	14.06	13	10.83	
	RCMI	59	46.09	51	42.50	
	NOT	2	1.56	0	0.00	

*LOS* indicates length of stay in hospital; *UEMI* indicates Urban Employee Medical Insurance; *URMI* indicates Urban Residents Medical Insurance; *RCMI* indicates Rural Cooperative Medical Insurance; *NOT* indicates no insurance

#### Comparative Analysis of QoL Before and After Treatment in the Groups

Patients’ physical and psychological conditions pre- and post- PC and CAT showed that the total QoL of both groups had improvements, as described in Table 2. The PC patients reported significant increases in their QoL score, from a pretreatment mean of 35.01 (SD = 4.66) to a post treatment mean of 41.83 (SD = 3.96) (t = -12.62, *p* < 0.05); the CAT patients also reported an increase in the QoL scores, with a pretreatment mean of 40.18 (SD = 2.81) and a post-treatment mean of 42.71 (SD = 3.88) (t = -4.55, *p* < 0.05). Comparison of the subitems' scores showed PC’s insignificant improvement in the 12 items' scores after treatments, including Appetite (t = -4.62, *p* < 0.05), Spirit (t = -7.78, *p* < 0.05), Sleep (t = -9.65, *p* < 0.05), Fatigue (t = -7.10, *p* < 0.05), Pain (t = -25.23, *p* < 0.05), Family care and understanding (t = -3.23, *p* < 0.05), Support from friends (t = -2.76, *p* < 0.05), Self-awareness of disease (t = -3.74, *p* < 0.05), Attitudes to therapy (t = -2.97, *p* < 0.05),Self-care ability (t = -4.02, *p* < 0.05), Side effects (t = -9.23, *p* < 0.05), and Countenance (t = -14.43, *p* < 0.05). While the patients in CATs, had significant improvements in Pain (t = -6.01, *p* < 0.05) and Side effects (t = -5.11, *p* < 0.05), another 10 items had no significant differences.

**Table 2:** Comparative analysis of QoL pre- and post therapy in PCs and CATs

Items		PCs				CATs			
		**Pretherapy (N = 128)**	**Posttherapy (N = 128)**	**t**	**P**	**Pretherapy (*N* = 120)**	**Posttherapy (*N* = 120)**	**t**	**P**
**QoL**		**35.01 ± 4.66**	**41.83 ± 3.96**	**-12.617**	**0**	**40.18 ± 2.81**	**42.71 ± 3.87**	**-4.548**	**0**
Physical condition	Appetite	2.55 ± 0.94	2.89 ± 0.86	-4.624	0	3.57 ± 0.69	3.64 ± 0.67	-0.493	0.626
	Sleep	3.04 ± 0.95	3.87 ± 0.63	-9.645	0	3.85 ± 0.44	3.92 ± 0.46	-0.812	0.424
	Fatigue	2.20 ± 0.84	2.67 ± 0.71	-7.099	0	2.86 ± 0.70	3.07 ± 0.85	-1.536	0.136
	Pain	2.16 ± 0.61	4.01 ± 0.44	-25.232	0	2.89 ± 0.41	3.78 ± 0.62	-6.011	0
	Self-care ability	2.28 ± 0.89	2.51 ± 0.86	-4.015	0	2.60 ± 0.56	2.71 ± 0.65	-0.769	0.449
	Side effects	2.76 ± 0.77	3.67 ± 0.52	-9.233	0	2.11 ± 0.31	2.71 ± 0.53	-5.109	0
	Countenance	2.57 ± 0.73	3.71 ± 0.48	-14.429	0	3.29 ± 0.53	3.50 ± 0.69	-1.652	0.11
Psycho-mental state	Spirit	2.46 ± 0.84	3.04 ± 0.76	-7.779	0	3.25 ± 0.51	3.46 ± 0.63	-1.996	0.056
	Self-awareness of disease	3.92 ± 0.50	4.08 ± 0.41	-3.742	0	4.18 ± 0.47	4.21 ± 0.49	-0.57	0.573
	Attitudes to therapy	3.87 ± 0.51	3.97 ± 0.45	-2.966	0.004	4.07 ± 0.37	4.10 ± 0.49	-0.57	0.573
Social relationships	Family care and understanding	3.78 ± 0.69	3.92 ± 0.57	-3.23	0.002	3.96 ± 0.33	3.92 ± 0.37	0.441	0.663
	Support from friends	3.40 ± 0.72	3.49 ± 0.75	-2.755	0.007	3.53 ± 0.50	3.64 ± 0.48	-1.362	0.184

#### Comparative Analysis of QoL Before and After Treatment Between the Groups

Independent samples tests were used to analyze the QoL of patients in the PC and CAT groups on the day of admission, and it was found that the mean QoL score of patients in PC was 35.01(SD = 4.66) and that of CAT patients was 40.18 (SD = 2.81). The PC patients' QoL was significantly lower than that of CAT patients (t = -10.50, *p* < 0.05). When Comparing the subitems before therapy, except for Side effects (2.76 ± 0.77 VS 2.11 ± 0.31, t = 6.12, *p* > 0.05), the other 11 items’ scores in PC were lower than those of CAT, and there were significant differences in 8 subitems, including Appetite (t = -6.04, *p* < 0.05), Spirit (t = -5.75, *p* < 0.05), Sleep (t = -5.94, *p* < 0.05), Fatigue(t = -3.66, *p* < 0.05), and Pain(t = -6.93, *p* < 0.05), as listed in Table 3.

**Table 3:** Comparative analysis of QoL pre- and post-therapy between PCs and CATs

Items		Pre-treatment				Post-treatment			
		**PCs**	**CATs**	**t**	**P**	**PCs**	**CATs**	**t**	**P**
		**(*N* = 128)**	**(*N* = 120)**			**(*N* = 128)**	**(*N* = 120)**		
QoL		35.01 ± 4.66	40.18 ± 2.81	-10.495	0	41.83 ± 3.96	42.71 ± 3.87	-1.014	0.313
Physical condition	Appetite	2.55 ± 0.94	3.57 ± 0.69	-6.038	0	2.90 ± 0.86	3.64 ± 0.67	-4.136	0
	Sleep	3.03 ± 0.95	3.85 ± 0.44	-5.942	0	3.87 ± 0.63	3.93 ± 0.46	-0.435	0.665
	Fatigue	2.21 ± 0.84	2.85 ± 0.70	-3.656	0	2.67 ± 0.71	3.07 ± 0.85	-2.435	0.017
	Pain	2.17 ± 0.61	2.89 ± 0.41	-6.927	0	4.01 ± 0.44	3.79 ± 0.62	1.759	0.087
	Self-care ability	2.28 ± 0.89	2.60 ± 0.56	-2.203	0.031	2.51 ± 0.86	2.71 ± 0.65	-1.121	0.265
	Side effects	2.76 ± 0.77	2.11 ± 0.31	6.118	0	3.67 ± 0.52	2.71 ± 0.53	8.18	0
	Countenance	2.58 ± 0.73	3.29 ± 0.53	-5.43	0	3.70 ± 0.48	3.50 ± 0.69	1.442	0.158
Psycho-mental state	Spirit	2.46 ± 0.84	3.25 ± 0.51	-5.749	0	3.04 ± 0.76	3.46 ± 0.63	-2.638	0.01
	Self-awareness of disease	3.92 ± 0.50	4.18 ± 0.47	-2.335	0.021	4.08 ± 0.41	4.21 ± 0.49	-1.302	0.2
	Attitudes to therapy	3.87 ± 0.51	4.07 ± 0.37	-1.864	0.065	3.97 ± 0.45	4.10 ± 0.49	-1.292	0.199
Social relationships	Family care and understanding	3.78 ± 0.69	3.96 ± 0.33	-1.811	0.073	3.92 ± 0.57	3.93 ± 0.38	-0.057	0.955
	Support from friends	3.40 ± 0.72	3.54 ± 0.50	-0.927	0.356	3.49 ± 0.75	3.64 ± 0.48	-1.241	0.219

After the treatments, the mean QoL of patients in PC was 41.83 (SD = 3.96), and that of patients in CAT was 42.71 (SD = 3.87); although the QoL score of PC was still lower than that in CAT, the difference did not reach statistical significance (*p* > 0.05). When Comparing the subitems on the day of discharge, for patients in PC, there were 8 subitems that were not significantly different from those in CAT, including Sleep (t = -0.44, *p* > 0.05), Pain (t = 1.76, *p* > 0.05), Family care and understanding (t = -0.06, *p* > 0.05), Support from friends(t = -1.24, *p* > 0.05), Self-awareness of disease (t = -1.30, *p* > 0.05), Attitudes to therapy (t = -1.29, *p* > 0.05), Self-care ability (t = -1.12, *p* > 0.05), and Countenance(t = 1.44, *p* > 0.05), while the side effects of patients in PC were significantly higher than those of patients in CAT (3.67 ± 0.52 VS 2.71 ± 0.53, t = 8.18, *p* < 0.05).

### Cost-utility analysis

#### QALYs

The importance of indicators in the CQLQ scale was evaluated through Delphi, and there were 33 responses. The Kendall Coefficient test used for the indexes of the two levels, which showed that the coordination coefficient of the first level was 0.78 (χ2 = 51.19, *p* < 0.001), and that of the second level was 0.664 (χ2 = 240.95, *p* < 0.001), indicating that experts' assessment of the importance of evaluation indicators tends to be consistent, as listed in Table 4.

**Table 4:** Evaluation of the importance of the indicators in the CQLQ

First level index	Weight	Second level index	Weight	Combination weight
**Physical condition**	0.5934	Appetite	0.0807	0.0479
		Sleep	0.1454	0.0863
		Fatigue	0.0581	0.0345
		Pain	0.3906	0.2318
		Self-care ability	0.1050	0.0623
		Side effects	0.1845	0.1095
		Countenance	0.0357	0.0212
**Psycho-mental state**	0.3157	Spirit	0.3009	0.0950
		Self-awareness of disease	0.3690	0.1165
		Attitudes to therapy	0.3301	0.1042
**Social relationships**	0.0908	Family care and understanding	0.8306	0.0755
		Support from friends	0.1694	0.0153

Analytic hierarchy process software Yaahp (V12.1) was used to construct the hierarchical model, and the weight of each item was calculated. We then calculated QALYs after weighing the items from the two groups of patients' QoL pre-and post- therapy. For patients in PC, the QALY (days) increased by 55.9 days, and for patients in CAT, the QALY increased by 24.0 days. The difference was statistically significant, as described in Table 5.

#### Expenses Analysis

The expenses analysis focused on the incremental medical expenses of the patients in PC relative to those in CAT. We gathered 4123 patient hospitalization expense reports(PC = 1391, CAT = 2732) from the electronic case database from January 1,2016, to August 31,2018, as listed in Table 6. The χ^2^ test for categorical variables showed that expenses were significantly different across patients' demographic and clinical factors, such as age, sex, LOS and type of disease. To control the influence of major confounding factors on the expenses, we used PSM 1:1 pairing for patients in PC and patients in CAT(caliper was ± 0.05) and successfully matched 613 pairs. On average, PC costs per patient were 13,743.5 RMB (SD = 11,574.1) versus 11,689.0 RMB (SD = 8876.8) for CAT. The average cost of patients in the PC group was significantly higher than that of those in the CAT group (t = 3.44, *p* < 0.05), as listed in Table 7. We compared the composition of average hospitalization expense, and found that, the average medical treatment expense(2153.2 ± 2290.1 VS 1678.8 ± 1733.4, t = 4.14, *p* < 0.001) and nursing expense(815.3 ± 695.8 VS 360.5 ± 432.1, t = 13.90 *p* < 0.001) of per patient in the PC were significantly higher than that in the CAT, and there was no significant difference in the average drug expense(6427.3 ± 5994.4 VS 6371.3 ± 5357.7, t = 0.16 *p* > 0.05), while the average imaging(250.9 ± 507.1 VS 547.7 ± 735.8, t = -8.22 *p* < 0.001) and laboratory expense(870 ± 708.5 VS 1127.3 ± 827.8, t = -5.73 *p* < 0.001) per patient in the PC was significantly lower than that in CAT.

**Table 5:** Comparative analysis of QALYs plus of PCs and CATs

Items	PCs	CATs	t	P
QALYs Plus	55.90 ± 18.80	24.00 ± 8.60	7.506	0.000﻿

**Table 6:** Patients' Hospitalization Expenses Analysis

Variable		n	Cost	F	P
Sex	Male	2482	11,589.4 ± 10,172.5	3.73	0.047
	Female	1641	12,217.9 ± 11,421.7		
Age	≤ 40	522	8728.4 ± 9090.6	17.98	0.000
	41—50	682	10,431.2 ± 9206.7		
	51—60	953	11,425 ± 9450.8		
	61—70	1131	12,038 ± 10,217.4		
	71—80	609	14,809.4 ± 13,210.2		
	> 80	226	16,030.9 ± 14,200.3		
LOS	≤ 3	281	3005.2 ± 1510.4	401.14	0.000
	4–7	1101	5563.2 ± 2782.8		
	8–14	1503	9702.6 ± 4293.4		
	15–30	998	18,013.8 ± 8353.2		
	> 30	240	38,711.3 ± 18,453.2		
Tumor site	Digestive	1812	12,207.8 ± 11,079.7	10.03	0.000
	Breast	866	12,838.3 ± 10,859.3		
	Head and neck	557	9650.6 ± 7485.7		
	Gynecological	212	14,372.7 ± 12,284.3		
	Others	182	9443.8 ± 7609.3		
	Genitourinary	131	15,326.6 ± 13,752.5		
	Central nervous	94	12,439.2 ± 11,850.7		
	Eye	85	2231.8 ± 1419.4		
	Musculoskeletal	75	12,321.9 ± 12,359.6		
	Lymphatic	48	10,396.2 ± 8864.6		
	Abdominal	45	13,415.1 ± 12,672.5		
	skin	16	13,471.1 ± 17,477.6		

*LOS* Length of stay in hospital

#### Comparison of cost-utility between groups

QALYs were used as a utility indicator to estimate the minimum allowable costs per QALYs, which revealed that patients in PC who earned one more day of QALYs had to pay 245.9RMB, while patients in CAT had to pay 487.0RMB. This finding suggested that under the assumption that advanced cancers had the same survival time, patients in PC paid higher expenses than patients in CAT, but from the "Maximum-Benefit" perspective, the expense per QALYs per patient was nearly 50% less, as shown in Table 7.

**Table 7:** Comparative analysis of Average Hospitalization Costs and Cost-Utility of PCs and CATs

Items	PCs	CATs	t	P
Average Hospitalization Costs	13743.5 ± 11574.1	11689.0 ± 8876.8	3.44	0.001
Cost-utility	246.8	487.0	-	-

## Discussion

This study analyzed QoL before and after treatments through intra- and inter-group comparisons and showed that although both groups had improved after treatments, PC had significant improvements in all subitems, while CAT had improvements only in items of Pain and Side effects. Besides, in the pretreatment stage, patients' QoL in the PC group was significantly lower than that in the CAT group, and in post-treatment stage, there was no significant difference in QoL between the two groups. There may be three reasons why PC has better clinical efficiency than CAT: (1) Rather than a focus on disease treatment as in CAT, PC pays more attention to patients' physical function, psychology and emotion, so patients with advanced cancer receiving PC experience remarkable effects in improving their QoL scores in their final stage of life. (2) The elderly and weaker patients may be more likely to opt for PC. Patients in PC and their families have lower expectations of therapy outcomes than patients in CAT and thus achieving higher scores, as reported in the results. (3) PC is mainly based on nursing services, psychological support and analgesic treatment, and patients maintain good, constant communication with medical teams, helping to improve patient satisfaction. These findings suggest that PC is a special kind of patient- and family-centered health care that focuses on effective management of symptoms and psychological support, which can help patients with terminal diseases obtain better QoL at their end-of-life. Thus, we need to promptly weaken the influence of traditional cultural values of “encourage efforts to heal rather than alleviate suffering”, and popularize the knowledge of PC at the public level. Let more eligible patients receive PC services earlier.

However, PC is usually regarded by patients and their families as “Abstention therapy”, or "Alternative treatments" [7]. Therefore, in the traditional impression, PC is often regarded as a cheaper treatment. However, this study found that PC is not a cheaper service; the differences in access criteria, paths of diagnosis and therapies, and post healing expectations between patients in PC and CAT determine the differences in outcomes and expenses. PC is not giving up treatment, it is necessary to reduce the pressure of the tumor on the patient's nerves and vital organs by using appropriate radiotherapy, chemotherapy and other conventional anti-cancer methods to prolong patients' survival time and to relieve all kinds of painful symptoms and help patients and their families face death with a peaceful and positive attitude, which makes the average expense, especially intervention and nursing expenses of PC even higher than that of CAT. Many previous studies have concluded that PC can effectively reduce the medical cost of patients [5, 29–36]. However, in this study, through paired comparison, we found that PC could reduce medical costs for patients not just in a simple comparison of average expenses but based on a comprehensive evaluation including expense and QALYs values of two factors. Under the medical insurance system of co-payment, the Cost-Utility analysis on the treatment methods can guide patient and insurance administration choose more effective services.

This research applied the method of health economics evaluation to link medical expense with treatment effect, which objectively and truly reflects the clinical efficacy and economic value of PC treatment. It is recommended that the combination of microscopic disease research and macrosystem construction, economic benefit and social benefit can provide a valuable reference in China to better formulate and improve relevant policies to promote the construction of a PC service system.

There are three limitations that should be noted. First, although patients with advanced cancer comprise the main part of PC services, patients receiving PC also include other terminal diseases, such as AIDS, motor neuron diseases, advanced heart failure, and advanced renal failure [37–39]. This study failed to analyze the effects of the treatments of terminal diseases other than cancer, thus, some limits raise the evaluation of PC to a more macroscopic level. Second, different age, cancer type and cancer stage may confound factors affecting the QoL scores comparison. Because small sample size obtained and the problem of information loss, we could not use PSM 1:1 pairing to eliminate the influence of confounding factors. Third, in this study, only one hospital was selected as the data collection point; although West China Fourth Hospital is a pioneering representative of China's PC service, because there was a single sampling source, the analysis results of the data are vulnerable to mixed factors such as hospital operation and patient source.

## Conclusion

For patients with advanced cancer, PC appears to have better clinical effects and cost-utility than the CAT. We believe that PC is an important proactive care strategy with specific clinical services that helps patients with terminal diseases have a better QoL in their final life-stage. Hence, China should actively promote the construction of hospital-based PC system to improve the cost and utility for patients and the whole society coping with incurable illness.

## Data Availability

The datasets used and analyzed during the current study are available from the corresponding author on reasonable request.

## Abbreviations

QoL: Quality of Life
PC: Palliative Care
CAT: Conventional Anticancer Treatment
CQLQ: Chinese Quality of Life Questionnaire
QALYs: Quality-adjusted Life Years
LOS: Length of Stay
PSM: Propensity Score Matching
